# Transient and Steady Pervaporation of 1-Butanol–Water Mixtures through a Poly[1-(Trimethylsilyl)-1-Propyne] (PTMSP) Membrane

**DOI:** 10.3390/polym11121943

**Published:** 2019-11-26

**Authors:** VSSL Prasad Talluri, Petra Patakova, Tomas Moucha, Ondrej Vopicka

**Affiliations:** 1 Department of Biotechnology, University of Chemistry and Technology, Prague, Technická 5, 166 28 Prague, Czech Republic; tallurip@vscht.cz (V.P.T.); patakovp@vscht.cz (P.P.); 2 Chemical Engineering Department, University of Chemistry and Technology, Prague, Technická 5, 166 28 Praha 6, Czech Republic; mouchat@vscht.cz; 3 Department of Physical Chemistry, University of Chemistry and Technology, Prague, Technická 5, 166 28 Prague, Czech Republic

**Keywords:** pervaporation, butanol, water, PTMSP, diffusivity, ageing

## Abstract

The transient and steady pervaporation of 1-butanol–water mixtures through a poly[1-(trimethylsilyl)-1-propyne] (PTMSP) membrane was studied to observe and elucidate the diffusion phenomena in this high-performing organophilic glassy polymer. Pervaporation was studied in a continuous sequence of experiments under conditions appropriate for the separation of bio-butanol from fermentation broths: feed concentrations of 1.5, 3.0 and 4.5 *w/w* % of 1-butanol in nutrient-containing (yeast extract) water, temperatures of 37, 50 and 63 °C, and a time period of 80 days. In addition, concentration polarization was assessed. As expected, the total flux and individual component permeabilities declined discernibly over the study period, while the separation factor (average β = 82) and selectivity towards 1-butanol (average α = 2.6) remained practically independent of the process conditions tested. Based on measurements of pervaporation transients, for which a new apparatus and model were developed, we found that the diffusivity of 1-butanol in PTMSP decreased over time due to aging and was comparable to that observed using microgravimetry in pure vapor in 1-butanol. Hence, despite the gradual loss of free volume of the aging polymer, the PTMSP membrane showed high and practically independent selectivity towards 1-butanol. Additionally, a new technique for the measurement and evaluation of pervaporation transients using Fourier transform infrared spectroscopy (FTIR) analysis of permeate was proposed and validated.

## 1. Introduction

After more than 100 years of history in industrial acetone–butanol–ethanol (ABE) fermentation, patented by Chaim Azriel Weizmann in 1915, 1-butanol is again considered a promising biofuel [1]. 1-butanol is a valuable solvent, energy carrier and chemical feedstock that is extensively used in various industries [1,2,3]. This compound has been considered to be a useful partial replacement for automotive fossil fuels as it has a higher energy content, can be blended with gasoline to high concentrations and has a lower vapor pressure than ethanol, which is the far more commonly used renewable oxygenated blend with gasoline [2,4,5,6,7]. For instance, the use of 1-butanol in the product portfolio of biorefineries has led to increased revenues in comparison to base scenarios, which can eventually help mitigate environmental impacts in economic terms [4]. Similar to ethanol, 1-butanol can be produced from renewable feedstocks by clostridial ABE fermentation [8,9,10,11], and separated from the medium by pervaporation. For example, pervaporation has been found to resolve the shortcomings of liquid–liquid extraction and perstraction methods [5]. However, clostridia require not only a carbon source for their growth and production, but also a nitrogen source. Yeast extract is one of the most commonly used nitrogen sources in fermentation media, containing amino acids, peptides, proteins, saccharides, lipids, vitamins and minerals, and is therefore frequently used in the production of bio-butanol [12]. Nevertheless, the presence of yeast extract creates a complex fermentation medium that could interfere with the separation of bio-butanol. Thus, experiments were performed to test its influence on pervaporation membranes.

The production of bio-butanol by clostridia is limited by its toxicity to living cells, which necessitates its efficient separation from the fermentation broth [13,14,15]; the highest 1-butanol titer reached by clostridia is about 20 g/l [16]. As demonstrated in the literature, 1-butanol production can be increased significantly by integration of fermentation with pervaporation [17,18,19,20]. For this purpose, 1-butanol-selective membranes were commonly prepared from polydimethylsiloxane (PDMS) [17,18,21,22], poly[1-(trimethylsilyl)-1-propyne] (PTMSP) [23,24,25,26,27,28], or from a polymer of intrinsic microporosity (PIM-1) [29,30,31]. To our knowledge, membranes based on PTMSP show among the highest separation factors and permeabilities (fluxes) for the removal of 1-butanol from water. These properties are counterbalanced by physical aging and the complex mechanism of mass transport [32,33] through this glassy polymer, which decomposes above 200 °C without showing glass transition [34]. Thus, we studied in detail the influence of process conditions and the principles of mass transport on PTMSP membranes.

To study transport mechanisms, we propose a new type of apparatus for the measurement of pervaporation transients. Despite the principal simplicity of the pervaporation measurements, studies of transient pervaporation are not abundant and, to our knowledge, have only been performed with ethanol, water and other compounds through zeolite membranes [35,36], and, more recently, through chitosan membranes [37]. The separation of methanol and ethanol have also been studied using polyacrylonitrile (PAN) membranes [38], aroma compounds through polymeric composites [39], and various volatile compounds through PDMS as part of the analytical method known as membrane introduction mass spectrometry (MIMS) [40,41]. Similar experiments have also been carried out on the transient permeation of vapor mixtures [42,43]. These methodologies are mostly based on mass spectrometry, which delivers rapid quantification provided that the sample is at low pressure, or repeated sampling into a chromatograph, which is more commonly used for slower transients. Hence, we propose the use of a newly developed Fourier transform infrared spectroscopy (FTIR) route consisting of gaseous mixtures of the permeate with the carrier gas at atmospheric pressure. While the classical methods based on mass spectroscopy or chromatography typically require samples in the form of low pressure gas (MS) or liquid (GC, MIMS), our method enables on-line detection of diffusing components in gaseous streams at a broad range of pressures, such as from absolute vacuum to atmospheric pressure. In order to validate the results, the classical measurement of transient vapor sorption microgravimetry [44,45] was carried out.

## 2. Materials and Methods

### 2.1. Materials

PTMSP was purchased from Gelest, Inc. (Morrisville, Philadelphia, PA, USA). Butan-1-ol (Penta, Prague, Czech Republic, min. 99%), helium (Siad Czech, Prague, Czech Republic, 4.8) nitrogen (Siad Czech, 4.0) and liquid nitrogen (Siad Czech) were used as received. Yeast extract was purchased from Merck (Kenilworth, NJ, USA). Physical properties of 1-butanol and water were taken from a database [46].

### 2.2. Membrane Preparation

PTMSP was dissolved in *tert*-butyl methyl ether (MTBE, Lach-ner, Neratovice, Czech Republic, min. 99.8%) and mixed with a magnetic stirrer for 30 min to form a homogenous 3 wt % solution. The membrane was fabricated by casting this solution onto a glass petri dish and the solvent was allowed to evaporate slowly over 3 days at room temperature. The membrane thickness was 46 ± 2 µm for pervaporation and 74 ± 11 µm for vapor sorption microgravimetry. The membrane was soaked in methanol (Penta, p.a.) overnight and then dried in ambient air prior to its use in order to rejuvenate its physical structure [47].

### 2.3. Pervaporation System

**Figure 1:**
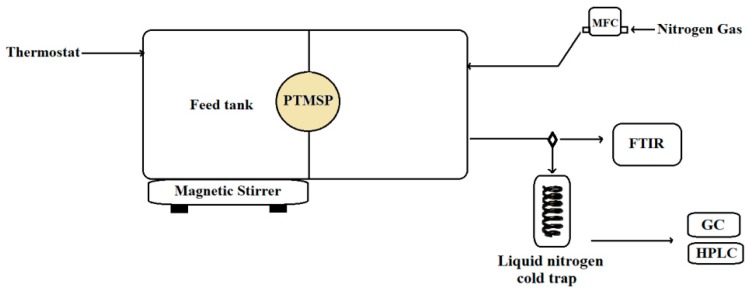
Schematic drawing of the pervaporation apparatus. MFC, mass flow controller; GC-MS, gas chromatograph with a mass spectrometric detector; HPLC, high performance liquid chromatography; FTIR, Fourier transform infrared spectroscopy.

Pervaporation (PV) experiments were conducted using a self-developed apparatus, the schematic diagram of which is shown in Figure 1. It consisted of two detachable parts made of duralumin EN AW 2007, between which the PTMSP membrane—having the effective area of 2.3 cm^2^—was placed; one part held the batch of the stirred feed solution at atmospheric pressure (average 98 kPa) and the second part provided the sweeping of the permeate with the sweep gas (nitrogen) at atmospheric pressure. The whole apparatus was maintained at a constant temperature using a water circulator (Huber Ministat 125, Berching, Germany). The optimum temperature for clostridial strains for the production of 1-butanol is 37 °C, so the ideal pervaporation temperature is 37 °C for in situ product recovery (ISPR) in continuous fermentation. In the case of batch fermentation, however, pervaporation can be operated at even higher temperatures. The experiments were therefore performed at 37, 50 and 63 °C. At each temperature, 1.5 g of 1-butanol was added three times into 100 mL of initially pure demineralized water (feed), thus creating cumulative totals of 1.5, 3.0 and 4.5 g of 1-butanol. In reality, 15 g/L is the highest relevant concentration for 1-butanol separation from ABE fermentation broth. Certain clostridial strains can produce a maximum concentration of 20–25 g/L of 1-butanol [16]. However, reaching this maximum 1-butanol concentration causes death of the microbial cells. Therefore, it is reasonable to separate 1-butanol from the medium when its concentration is lower than 15 g/L during continuous or fed-batch ABE fermentation. Relieving product toxicity may also improve 1-butanol production during fermentation. The pervaporative separation of more concentrated feeds is thus more relevant to the post-processing of liquors produced by batch fermentation. To simulate the culture broth, pervaporation experiments were repeated with the same mixtures enriched with 200 mg of yeast extract.

During the pervaporation runs, the feed solution was stirred with a magnetic stirrer (250 rpm). The hydrodynamic constraints near the membrane were estimated to be a Reynolds number of about 3000, which corresponds with a transient (not fully turbulent) regime, resulting in a mass transfer coefficient on the retentate side of the membrane of around 5 × 10^−5^ m⋅s^−1^, calculated using the estimate of a liquid–solid mass transfer coefficient by Barker and Treybal [48]. Under these conditions, minor resistance of the liquid film against the overall mass transfer through the membrane was observed, which will be shown in the Results. Therefore, the concentration polarization of the membrane was neglected. Nitrogen was used as the sweep gas at a constant flow rate of 75 cm^3^ STP⋅min^−1^ using a mass flow controller (DFC 26, Aalborg, Orangeburg, NY, USA). The permeate was collected in a liquid nitrogen trap, which was hermetically closed prior to weighing; the net permeate weight was determined using an Ohaus DV215CD balance (Nänikon, Switzerland) [49]. The molar fraction of 1-butanol in the feed and permeate was analyzed by means of gas chromatography or, in the case of experiments with yeast extract, by means of high-performance liquid chromatography (HPLC).

### 2.4. Vapor Sorption Microgravimetry

The sorption of 1-butanol vapor in PTMSP was measured as previously described [44] using a gravimetric sorption apparatus equipped with a quartz spiral microbalance. Due to limitations of the pressure transducers, the device was not used above 37 °C. Sorption experiments were initiated by conducting the vapor of 1-butanol to the previously evacuated chamber containing the degassed PTMSP film. The sorption uptake was continuously measured until equilibrium was reached, and constant pressure of 1-butanol vapor was maintained. As diffusion in PTMSP is known to be relatively fast and the release of the vapor was not instantaneous, the diffusion coefficient was calculated from the kinetics of the transient sorption data and the actual pressure of 1-butanol vapor using Equations 10, 13 and 14 from the literature [45].

### 2.5. Gas Chromatography Analysis

The concentration of 1-butanol in the feed and permeate was determined by gas chromatography with a polar stationary phase in a capillary column (Elite-WAX, Perkin Elmer, Waltham, MA, USA). The GC-MS system used in this study consisted of a quadrupole instrument with a direct capillary column interface and an electron-ionization type ion source (Clarus 500, Perkin Elmer-Arnell); helium was used as the carrier gas. The mass spectrometer with quadrupole detector was used in the selected ions monitoring mode; molecular ion 18 Da was used for water, and ions 41 Da and 56 Da for 1-butanol. The chromatograph was calibrated prior to measurements.

### 2.6. HPLC Analysis

The concentration of 1-butanol in the feed containing yeast extract was determined by HPLC (Agilent series 1200, Santa Clara, CA, USA) under the following conditions: mobile phase 5 mM H_2_SO_4_, flow rate 1 mL/min, column temperature 60 °C, injection volume 20 µL, stationary phase IEX H polymer (Watrex, Prague, Czech Republic), and refractive index detection.

### 2.7. SEM Analysis

The PTMSP membrane was inserted in an upright position in a mold that was filled with castable cold mounting compound (Varidur 200 from Buehler, Lake Bluff, IL, USA). After hardening, the samples were ground with a Buehler grinder polisher at 220 rpm with increasingly fine grades of abrasive paper (grit size 400, 800, 1200 and 2500). The samples were coated with a thin layer of gold to prevent charging. The cross-section of the PTMSP membrane was characterized using a scanning electron microscope (SEM; Tescan VEGA 3-LMU, 20 kV, Brno, Czech Republic).

### 2.8. Contact Angle Measurement

The hydrophobicity of the new and aged membrane was studied by measuring the static contact angle (θ) of a sessile water droplet using a contact angle meter (OneAttension Theta, Biolin Scientific, Stockholm, Sweden). The contact angle was determined by OneAttension 3.0 software. Before the measurement, the membrane was cleaned with methanol to degrease its surface. A water droplet with a volume of 2 µL was placed on the surface of the membrane fixed to a glass slide and an image of the water drop was captured using a high definition camera. The contact angle was defined by fitting the Young–Laplace equation around the droplet using the system’s software.

### 2.9. Measurement of Transient Pervaporation

After the concentration of 1-butanol in the feed was changed, the stream of nitrogen containing the permeate escaping the pervaporation unit was continuously analyzed using an FTIR spectrometer (iS10, Thermo Fisher Scientific Inc., Waltham, MA, USA) equipped with a gas cell maintained at 48 °C and a mercury cadmium telluride (MCT-A) detector. The approximate volume of the gas cell was 0.25 dm^3^ [50] and the nitrogen flow rate was 75 cm^3^ (STP) min^−1^. The intensities of the selected compound-specific bands in the gaseous mixture were measured using the Omnic 8 software (Thermo Fisher Scientific Inc., Waltham, MA, USA); four scans were taken for one spectrum at a resolution of 0.5 cm^−1^ in a time-series of 30 minutes. Bands ranging from 974.88 to 1146.76 cm^−1^ (1-butanol) were used for the analysis (see Supplementary Data for the spectrum).

**Figure 2:**
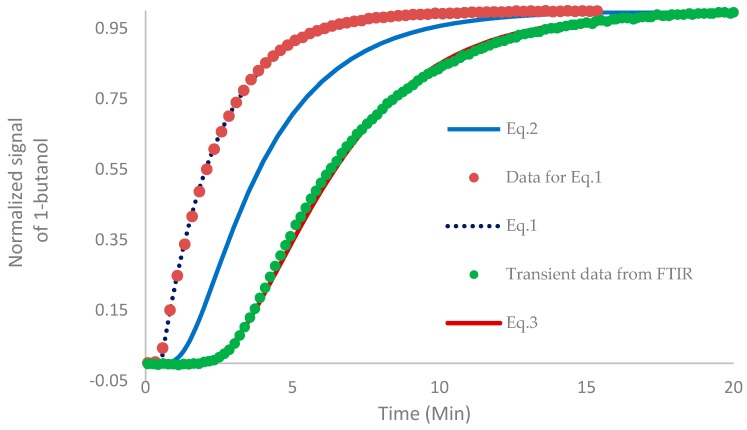
Normalized response of the analyzer (FTIR, band 974.88–1146.76 cm^−1^) to the unit step of permeate concentration approximated with Equation (1). Points represent measured data, curves represent models—Equation (1), Equation (2) and Equation (3). Pervaporation through poly[1-(trimethylsilyl)-1-propyne] (PTMSP), 1-butanol concentration step 0–1.5 g/100 g of water at 37 °C.

As the volume of the gas cell of the measuring device was not negligible, the sample washing out distorted the concentration–time profiles measured. Hence, the dynamic properties of the gas cell itself were tested by following the response of the detector to stepwise changes in permeate concentration under the same conditions used during the experiment. The response is shown in Figure 2 and was well parameterized with the one time constant model:

(1) y=1−exp[−a(t−b)]

in which *a* = 0.526 ± 0.001 min^−1^, the reciprocal time constant, and *b* = 0.50 ± 0.04 min, the delay caused by the piping.

The evolution of the flux of a component from a flat membrane, which is initially free of the diffusing compound, upon a sudden change of feed concentration, can be expressed as [51]:

(2) $$\frac{J_{i}}{J_{\infty }}=1+2\times \overset{\infty }{\underset{n=1}{\sum }}{\left(-1\right)}^{n}\exp\left(-\frac{n^{2}\pi^{2}Dt}{l^{2}}\right)$$

in which *D* is the diffusion coefficient and *l* the membrane thickness. In this work, the first 15 summation terms were used due to the fast convergence of the sum. Clearly, Equation (2) can be used, in our case, for the analysis of the pervaporation transient of 1-butanol upon its first addition into initially pure water—i.e., upon the change of 1-butanol concentration from 0 to 1.5 *w/w* % as the resistance of the liquid film was negligible (see above). Despite solutions of Fick’s second law for non-uniform initial distribution being derived [52], the actual initial distribution depends, in the case of our experimental procedure, on the concentration dependence of the diffusivity, which is typically not known. Indeed, PTMSP is known to show highly anomalous sorption, swelling and diffusion [32,33,34,53]. We thus limited ourselves to studying the transient pervaporation of 1-butanol upon its first introduction into initially pure water (feed), i.e., to concentrations appropriate for continuous ISPR, and to comparing the retrieved diffusivity of 1-butanol with that determined from the measurement [44,45] of the kinetics of transient sorption of 1-butanol vapor in an initially degassed slab of the polymer.

The model signal given by Equation (2), which was modulated by a system having the step response given by Equation (1), can thus be expressed for *t* > *b* similar to previous works [45,54] and is commonly used in the analysis of heat transfer in calorimeters [55] by using Laplace and inverse Laplace transforms [56,57] and the definition of convolution, such that:

(3) $$\frac{J_{i}}{J_{\infty }}=1-\exp\left[-a\left(t-b\right)\right]+2\times \overset{\infty }{\underset{n=1}{\sum }}{\left(-1\right)}^{n}\times \frac{a\exp\left[-a\left(t-b\right)\right]+\left\{k\left[1-\exp\left(ab\right)\right]-a\right\}\exp\left(-kt\right)}{k-a}$$

in which

(4) $$k=\frac{n^{2}\pi^{2}D}{l^{2}}$$

The model accounting for the inertia of the analyzer given by Equation (1) therefore has the form of Equation (3). These models are plotted in Figure 2, together with the dependence theoretically observable with a detector showing no inertia and time delay (Equation (2)).

### 2.10. Measurement of Steady Pervaporation

The steady total permeate flux was determined by weighing the permeate collected over a certain time, whereby:

*J* = *m*/(*A t*) (5)

where *m* is the weight of the collected permeate, *A* the membrane area and *t* the time. The separation performance of pervaporation was expressed as the separation factor:

(6) $$\beta =\frac{x_{B,l}^{\;}\;/x_{W,l}^{\;}}{x_{B,0}^{\;}\;/x_{W,0}^{\;}}$$

where $x_{i,l}^{\;}$ and $x_{i,0}^{\;}$ stand for the molar fractions of the respective compounds (1-butanol, water) in the permeate and feed mixtures, respectively.

Besides using total flux and the separation factor, membrane efficiency can be expressed using the pervaporation separation index (PSI) defined by the following equation:

PSI = *J* (β − 1) (7)

Based on the solution–diffusion model, Baker et al. recently formulated a preferred way of reporting pervaporation data [58]. According to their approach, the flux of individual components across the membrane can be described as follows:

(8) $$j_{i}=\frac{P_{i}^{.}}{l}\left(\gamma_{i_{o}}^{L}x_{i_{o}}^{L}p_{i_{o}}^{\mathrm{sat}.}-p_{i}{}_{l}\right)$$

where *j_i_* is the partial flux of component *i*, $\gamma_{i_{o}}^{L}$ is the activity coefficient of component *i* in the liquid feed (denoted by subscript 0), $x_{i_{o}}^{L}$ is the mole fraction of the component *i* in the liquid feed, $p_{i_{o}}^{\mathrm{sat}.}$ is the pure component vapor pressure, $p_{i}{}_{l}$ is the partial pressure at the permeate (*l*) face of the membrane having the thickness *l*, and *P_i_* is the gas permeability of the membrane for the component *i*. Moreover, permeability can be expected as the product of the compound diffusivity and solubility in the material of the membrane.

Since the separation factor reflects not only the material properties of the membrane, but also of the entire experimental setup, material properties of different membranes can be compared using selectivity [58]:

(9) $$\alpha =\frac{P_{B}}{P_{W}}$$

for which permeability in mole-based units has to be used in order to obtain selectivity for a non-selective membrane. Clearly, the solubility coefficient can be calculated as the ratio of permeability and diffusivity. The thermodynamics of water–1-butanol solutions, which are used in Equation (8), were modelled using the Non Random Two Liquid (NRTL) model [59] with parameters taken from the literature [60]. Relative uncertainties of the evaluated quantities were assessed using the law of uncertainty propagation.

Low alcohols are known to show highly anomalous sorption isotherms in PTMSP [44,61]; the vapor does not practically sorb until a threshold vapor activity is reached. A model based on the Guggenheim–Anderson–De Boer (GAB) model of multilayer adsorption was previously proposed [44] to parameterize sorption using four adjustable parameters:

(10) $$v=\frac{v_{m}.h.ʄ.a}{\left(1-ʄ.a\right)\left(1-ʄ.a+h.ʄ.a\right)},\;h=B_{h}\exp\left(ɳa\right)$$

where v represents the amount of sorbate in the sorbent, $v_{m}$ stands for the capacity of the (first) adsorption mono layer of the sorbent, h is the ratio between the strength of binding of the molecules to the surface in the first layer and higher layers, ʄ is a parameter representing the ratio of the pressure of saturated vapors and a reference pressure, and a represents activity: *a*=*p*/*p*^sat^. The diffusion coefficient can be evaluated from the kinetics of the transient sorption of a vapor in a slab of polymer from the solution according to Fick’s second law; the actual model used in this work is described by Equations (10), (13) and (14) from the literature [52].

## 3. Results and Discussion

Pervaporation was studied in a continuous sequence of experiments under certain conditions, including those relevant to the separation of bio-butanol from fermentation broths. Specifically, the experiments comprised three subsequent additions of 1-butanol into water at a constant temperature, thereby achieving feed concentrations of 1.5, 3.0 and 4.5 *w/w* %; the membrane was repeatedly cleared of 1-butanol before each subsequent experiment by replacing the previously used feed with water. Pervaporation was studied at three temperatures, 37, 50 and 63 °C, with and without yeast extract, and over a time period of 80 days after the membrane was soaked in methanol.

Based on the law of propagation of uncertainty, the average relative uncertainties of the total flux, water permeability, 1-butanol permeability and butanol diffusivity were 15%, 20%, 25% and 10%, respectively. Consequently, the relative uncertainties of α, β, PSI and *S* were estimated based on the uncertainty propagation as 45%, 40%, 55% and 35%, respectively.

**Figure 3:**
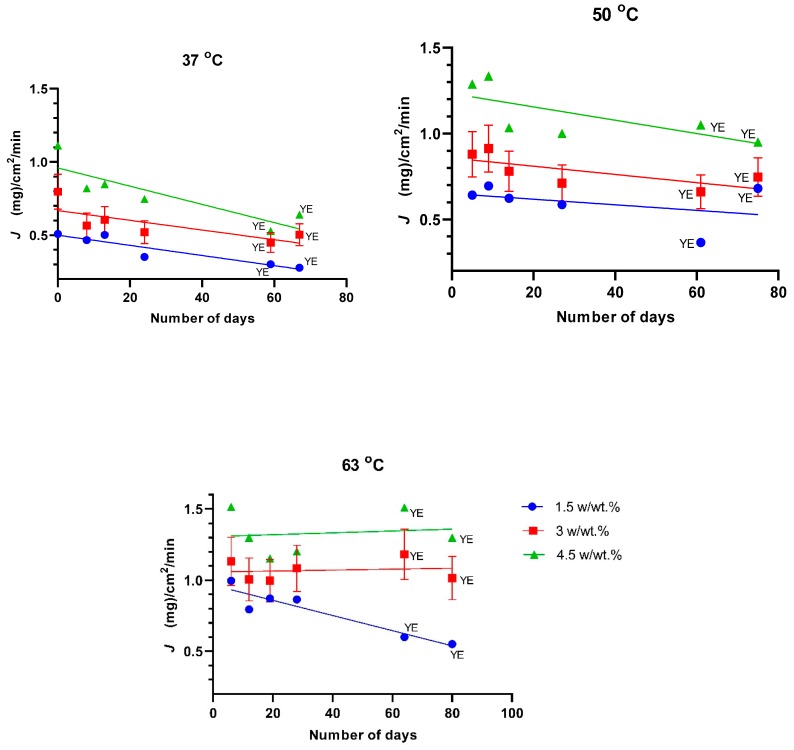
Total flux (*J*) at different feed concentrations against operating time. Colors at 37 °C and 50 °C have the same meaning as at 63 °C, feed concentrations were 1.5, 3.0 and 4.5 *w/w* % of 1-butanol. YE represents yeast extract, set to 200 mg/100 mL of water in the feed.

PTMSP is a glassy polymer with the highest known free volume fraction (up to 34%) [62]. The gradual collapse of the free volume of this polymer is commonly termed physical aging, although the physical structure can be restored by soaking it in liquid methanol [47]. Within the tested time period of 28 days (without yeast extract) and up to 80 days (with yeast extract) after being soaked in methanol (day zero), the total permeate fluxes decreased by approximately 50%, particularly in the case of experiments at 37 °C, while lesser decreases were observed in experiments with more concentrated feeds and at higher temperatures (Figure 3). Hence, aging was presumably counterbalanced to a certain extent by plasticization of the polymer for feeds which were more concentrated and at higher temperatures. A similar influence of polymer plasticization, i.e., expansion of free volume voids within the polymer due to solvent, has previously been observed for PTMSP treated with supercritical carbon dioxide [63].

The total fluxes observed in this work agreed with those reported in the literature [25,28]: 1.33 mg⋅cm^−2^⋅min^−1^ and 1.72 mg⋅cm^−2^⋅min^−1^ and separation factors of 135 and 70 were observed for PTMSP membranes having thicknesses of 16 and 22 µm for feed concentrations of 2 and 1.5 % *w/w* 1-butanol/water at 37 and 70 °C, respectively. Similar to these data, Masuda et al. [64] reported no change in the permeability of a PTMSP membrane for a 10 wt % ethanol/water feed over a time-span of 40 h at 30 °C.

**Figure 4:**
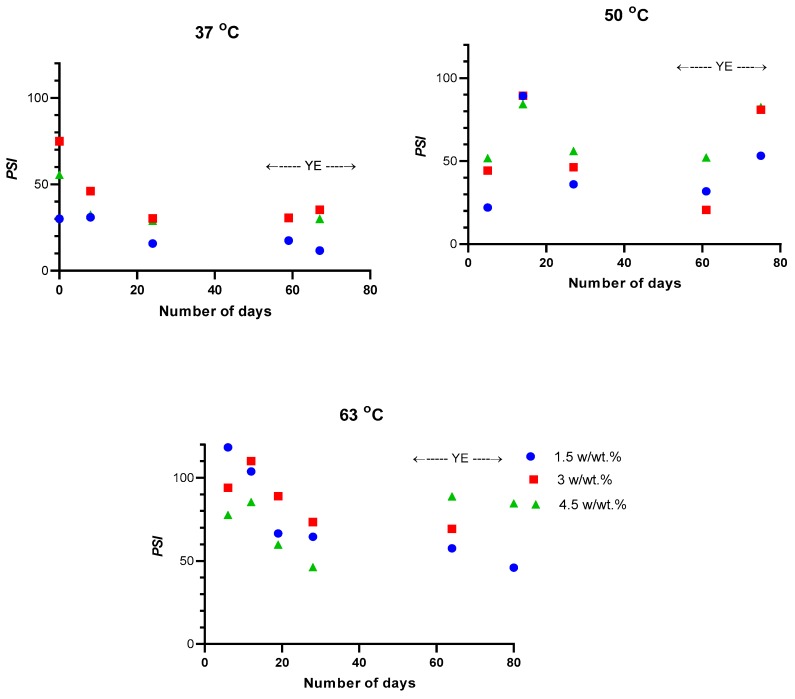
Pervaporation separation index (PSI) at different feed concentrations against operating time. Colors at 37 °C and 50 °C have the same meaning as at 63 °C. YE represents yeast extract, set to 200 mg/100 mL of water in the feed.

The effects of aging, temperature and feed concentration on the overall pervaporation performance expressed as PSI are shown in Figure 4. The highest PSI occurred at the highest investigated temperature (63 °C) and at the lowest investigated concentration (1.5 *w/w* %) of 1-butanol in the feed (Figure 4). PSI dropped by approximately 50% of the initial value at these conditions due to aging over the duration of the measurement. The presence of the yeast extract had only a minor influence at all inspected conditions.

As described above, membrane polarization was neglected due to low mass transfer resistance on the liquid retentate side of the membrane. As evident from the following data, the typical 1-butanol fluxes (flow intensities) through the membrane were approximately 0.8 mg⋅cm^−2^⋅min^−1^ (which corresponds to 0.48 kg⋅m^−2^⋅h^−1^) at 3 *w/w* % of butanol in the feed (which represents a 1-butanol concentration in the feed of 30 kg⋅m^−3^) and gave the following apparent permeability: *P*_A_ = (flux)/(driving force) = 4.44 × 10^−6^ m⋅s^−1^. The estimated mass transfer coefficient at the liquid retentate side of the membrane was 5 × 10^−5^ m⋅s^−1^. By comparing the mass transfer resistance on the liquid retentate side of the membrane, 1/*k* = 1/(5 × 10^−5^) s⋅m^−1^, with the total apparent mass transfer resistance, 1/*P*_A_ = 1/(4.44 × 10^−6^) s⋅m^−1^, we observed that the liquid side mass transfer resistance was one order of magnitude lower. More specifically, the liquid side mass transfer resistance contributed to around 8% of the total resistance. Neglecting the liquid film effect was acceptable given the range of experimental uncertainties.

**Figure 5:**
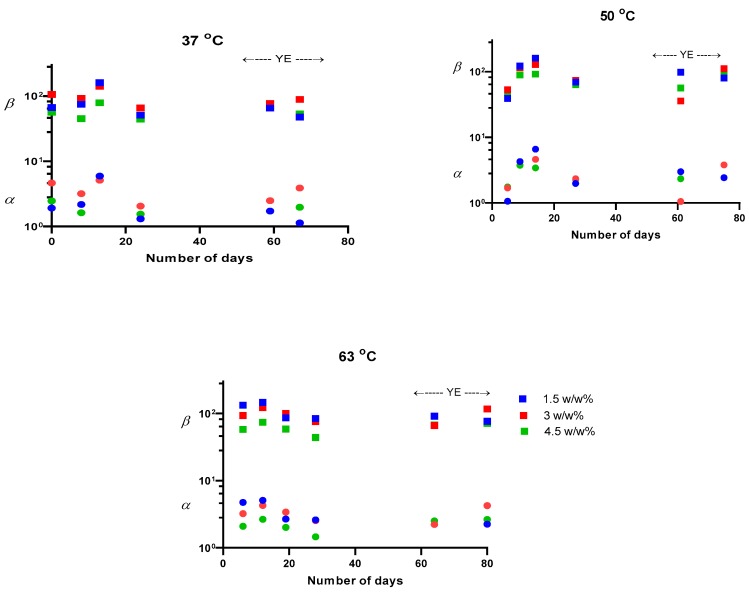
The selectivity (α) and separation factor (β) for 1-butanol using a PTMSP membrane, plotted against the different feed concentrations. Colors for 37 °C and 50 °C have the same meaning as for 63 °C. YE represents yeast extract, set to 200 mg/100 mL of water in feed.

Besides the aging of PTMSP, the high chance of fouling has commonly been of concern when a PTMSP membrane is to be used with real ABE fermentation broths containing living cells, sugars, and fatty acids etc. [26,65]. Yeast extract has commonly been used in the fermentation medium during the production of bio-butanol through ABE fermentation, as it is a source of vitamins, minerals and amino acids for microbial cells [12,66]. This component was thus added into the feeds in the ratio of 200 mg per 100 mL of water; no effect of the yeast extract on total flux was discerned (Figure 3). Neither the feed concentration of 1-butanol nor temperature, time or the presence of yeast extract was found to substantially influence the separation factor of pervaporation (β) or selectivity (α) of the PTMSP membrane (Figure 5). By comparing the average separation factor (β = 82) and selectivity (α = 2.6) and examining Equations (6,8,9), it follows that the organophilic pervaporation of the mixtures of 1-butanol with water appear to be positively influenced by the thermodynamic non-ideality of the solution to a significant extent.

**Figure 6:**
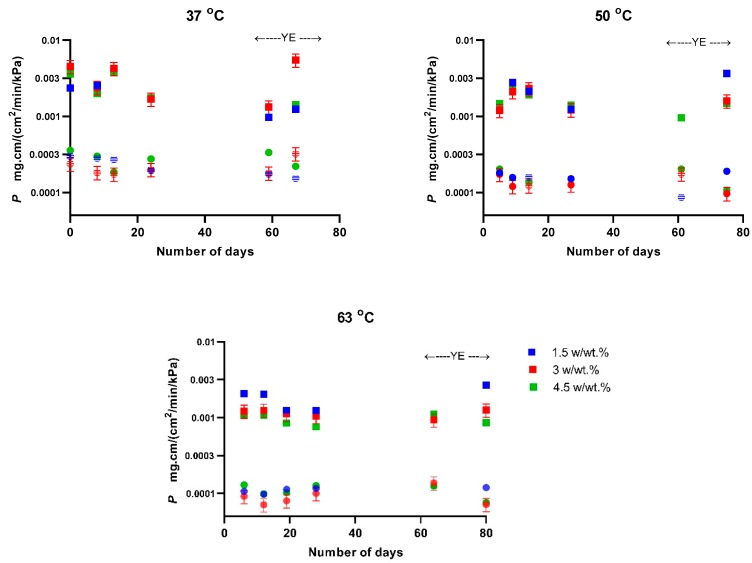
Permeability of PTMSP for 1-butanol (squares) and water (circles) at different feed concentrations plotted against operation time. Colors in the figures showing data at 37 °C and 50 °C have the same meaning as that in the figure for 63 °C, feed concentrations were 1.5, 3.0 and 4.5 *w/w* % of 1-butanol. YE represents yeast extract, set to 200 mg/100 mL of water in the feed.

The separation factor and selectivity showed minor dependences on temperature, feed concentration and polymer aging (Figure 6). At the same time, permeability of both 1-butanol and water decreased notably with increasing temperature, while their ratio (selectivity) remained constant. Mainly as a natural consequence of the temperature dependence of parameters in Equation (8), the total permeate fluxes were temperature dependent and were, on average, 1.2 times higher at 63 °C than at 37 °C (Figure S1).

**Figure 7:**
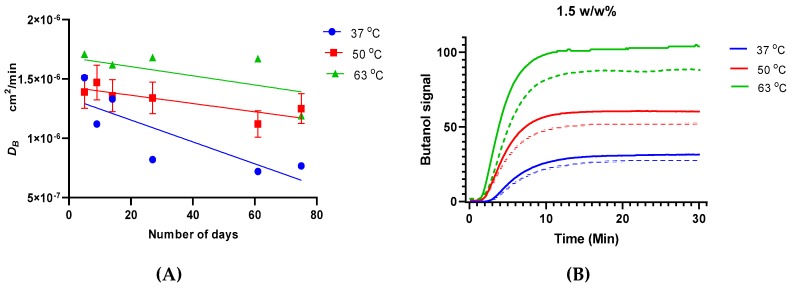
(**A**) Diffusivity of 1-butanol at different temperatures as a function of operation time at 1.5 *w/w* %; YE represents yeast extract, set to 200 mg/100 mL of water in the feed. (**B**) Pervaporation transients for the concentration change of 0–1.5 *w/w* % at different temperatures; solid and dotted curves are experimental data with and without yeast extract, respectively.

Diffusivity of 1-butanol, as evaluated from the pervaporation transients (Figure 7), was about one half of that evaluated from the 1-butanol vapor sorption transients (see supplementary Materials, Figure S3, average 2.9 × 10^−6^ cm^2^⋅min^−1^ at 37 °C). This difference possibly originates from the fact that the time of the initial mixing of the feed and the influence of the liquid film on the pervaporation transient were neglected. Despite that, however, it is clear that the presence of water did not significantly influence 1-butanol diffusivity in PTMSP in the case of pervaporation. Similar to the total pervaporation fluxes, which appeared to be mostly influenced by polymer aging in the case of the experiments conducted with diluted feeds (1.5 *w/w* % of 1-butanol) at low temperature (37 °C) (Figure 3), the diffusivities of 1-butanol in PTMSP were also mostly influenced by aging at low temperature (Figure 7). This was presumably due to the fact that decay of the free volume voids was not counterbalanced by plasticization of the polymer under these conditions. As permeability of both components decreased with increasing temperature (Figure 6), diffusivity of 1-butanol increased (Figure 7). Further, a decrease of solubility rather than a loss of free volume occurred due to increasing temperature.

The SEM micrographs and contact angle images for the rejuvenated membrane (Day 1) and aged membrane (Day 80) are shown in Supplementary Materials (Figures S5 and S6). No structural changes were observed on the cross-sectional surface of the membrane, whereas a minor decrease in hydrophobicity from the new (θ = 91) to the aged membrane (θ = 86) was noted. The decrease in 1-butanol diffusivity by approximately 50% over the time period tested was commensurate with that of the total flux in experiments with a diluted feed at 37 °C. As diffusivity is related to the free volume of the polymer, this confirms the physical nature of the aging of PTMSP.

The pervaporation transients observed for the experiments at higher temperatures were more rapid. The addition of yeast extract did not result in significant changes in the transients, while the effect of aging of the polymer was more pronounced (Figure 7). Consistent with that, the temperature dependence of 1-butanol diffusivity in PTMSP followed the Arrhenius type of dependence, yielding, on average, an activation energy of 1-butanol diffusion of 20.8 kJ⋅mol^−1^ (Supplementary Materials, Figure S2).

**Figure 8:**
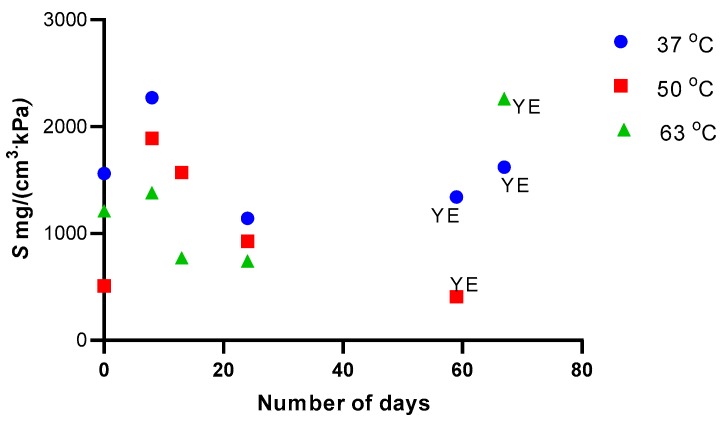
The solubility coefficient for 1-butanol in PTMSP evaluated for the 1-butanol concentration of 1.5 *w/w* % in the feed. YE represents yeast extract, dosed at 200 mg/100 mL of water in the feed.

**Figure 9:**
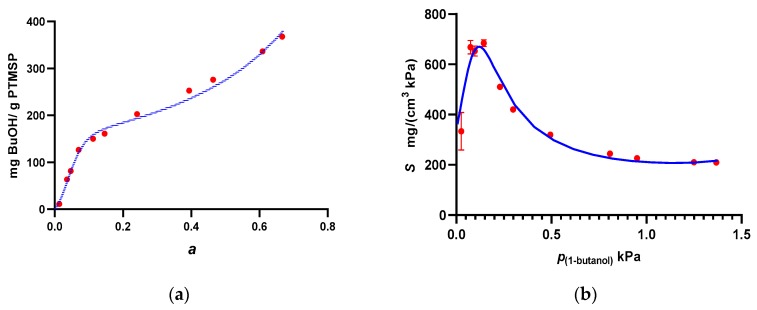
Sorption isotherm of 1-butanol vapor in PTMSP at 37 °C expressed as sorption uptake (**a**) and as sorption coefficient (**b**). Dots represent experimental data measured by means of single vapor sorption microgravimetry at 37 °C, curves represent the Guggenheim–Anderson–De Boer (GAB) model (Equation 10).

The solubility of 1-butanol in PTMSP during pervaporation was calculated by dividing permeability, as obtained from Equation (8), by diffusivity, as obtained from the pervaporation transients according to Equation (3). The solubility of 1-butanol in PTMSP decreased with increasing temperature and with time due to aging (Figure 8). The decrease of solubility with increasing temperature is consistent with the view that 1-butanol adsorbs or condenses in the free volume voids of bulk PTMSP. In order to compare the solubility calculated from pervaporation data using the solution–diffusion model, vapor sorption microgravimetry with 1-butanol vapor was performed. The uncertainty of the sorption uptake was approximately $2.5\;\mathrm{mg}\cdot g^{-1}$, while the relative uncertainty of diffusivity was 30%. The sorption isotherm showed a similar anomalous shape as those observed earlier for other low alcohols [44,61] and was well parameterized with Equation (10), yielding $v_{m}=152\;\mathrm{mg}\cdot g^{-1}$, $B_{h}=6.52$, ɳ=27.5, and *f* = 0.898 (Figure 9). The sorption coefficient, which is the ratio of sorption uptake and pressure, was then calculated from the sorption isotherm using the density of PTMSP, 0.78 $g\cdot {\mathrm{cm}}^{3}$ [32]. Clearly, the sorption coefficient evaluated from the pervaporation measurement was approximately twice as high as that observed from the vapor sorption microgravimetry. Hence, either diffusivity evaluated based on the pervaporation was lower than the real diffusivity due to the applied simplifying assumptions (the influence of the liquid film and of feed mixing were assumed to be negligible) or water influenced sorption and/or diffusivity of 1-butanol in PTMSP. Despite this difference, however, the solubility and diffusivity of 1-butanol in PTMSP were well commeasurable. Hence, the transport of the more sorbing component, 1-butanol, was not significantly influenced by the simultaneous transport of water during pervaporation.

## 4. Conclusions

In the present work, the transient and steady state pervaporation of 1-butanol–water mixtures through a PTMSP membrane was studied in a continuous series of experiments over a time period of 80 days under the following conditions: 1.5–4.5 *w/w* % of 1-butanol, 37–63 °C, and with or without fermentation nutrients (yeast extract). While the total flux and the individual component permeabilities decreased with time to a limited extent, the separation factor (β = 82) and selectivity (α = 2.6) appeared practically independent of the process conditions. The use of a new apparatus and a model for the evaluation of pervaporation transients enabled us to evaluate the diffusivity of 1-butanol during pervaporation; diffusivity decreased over time due to aging of the polymer and increased with temperature. Aging of the polymer was most pronounced for experiments with a diluted feed (1.5 *w/w* %) and low temperature (37 °C), while it was counterbalanced, to some extent, by plasticization of the polymer with 1-butanol and more concentrated feeds at higher temperatures. The new method of measuring steady and transient pervaporation enabled us to determine diffusivity of 1-butanol in PTMSP and comparison of this quantity with an independent method—vapor sorption microgravimetry. A good agreement between the solubility and diffusivity of 1-butanol was observed using these two independent measurements, thereby validating the method and illustrating that transport of the highly sorbing species, 1-butanol, was not significantly influenced by the simultaneous transport of water through the PTMSP membranes.

## Supplementary Materials

The following are available online at https://www.mdpi.com/2073-4360/11/12/1943/s1. Figure S1: Total flux (*J*) at different temperatures as a function of operating temperature. YE is yeast extract at 200 mg/100 mL water in the feed., Figure S2: Temperature dependence of 1-butanol diffusivity in PTMSP. YE represents yeast extract dosed at 200 mg/100 mL of water in the feed. The black line represents Arrhenius-type model data for all feeds without yeast extract and red lines the model for feeds with yeast extract., Figure S3: Diffusivity of 1-butanol vapors in PTMSP as measured with single vapor microgravimetry at 37 °C., Figure S4: FTIR spectra for pure 1-butanol and water vapors. Bands from 1146.76 to 974.88 cm^−1^ were used to follow 1-butanol., Figure S5: Cross sectional scanning electron microscope images of aged membrane and fresh PTMSP membrane with high magnification 5000 times (a) Aged (b) fresh membrane., Figure S6: Contact angle measurement images of fresh and aged membrane PTMSP membrane (a) Fresh membrane (b) aged membrane.
