# Supplementary material for: Transient and Steady Pervaporation of 1-Butanol–Water Mixtures through a Poly[1-(Trimethylsilyl)-1-Propyne] (PTMSP) Membrane
Source: Polymers (Basel). 2019 Nov 26;11(12):1943. doi: 10.3390/polym11121943 (PMC6960892; doi:10.3390/polym11121943)
Supplement: Supplementary file 1 [file polymers-11-01943-s001.pdf]

polymers

MDPI

Supplementary Materials: Transient and Steady Pervaporation of 1-Butanol–Water Mixtures through a Poly[1-(Trimethylsilyl)-1-Propyne] (PTMSP) Membrane

VSSL Prasad Talluri, Petra Patakova, Tomas Moucha and Ondrej Vopicka

The temperature dependence of total flux is shown in Figure S1.

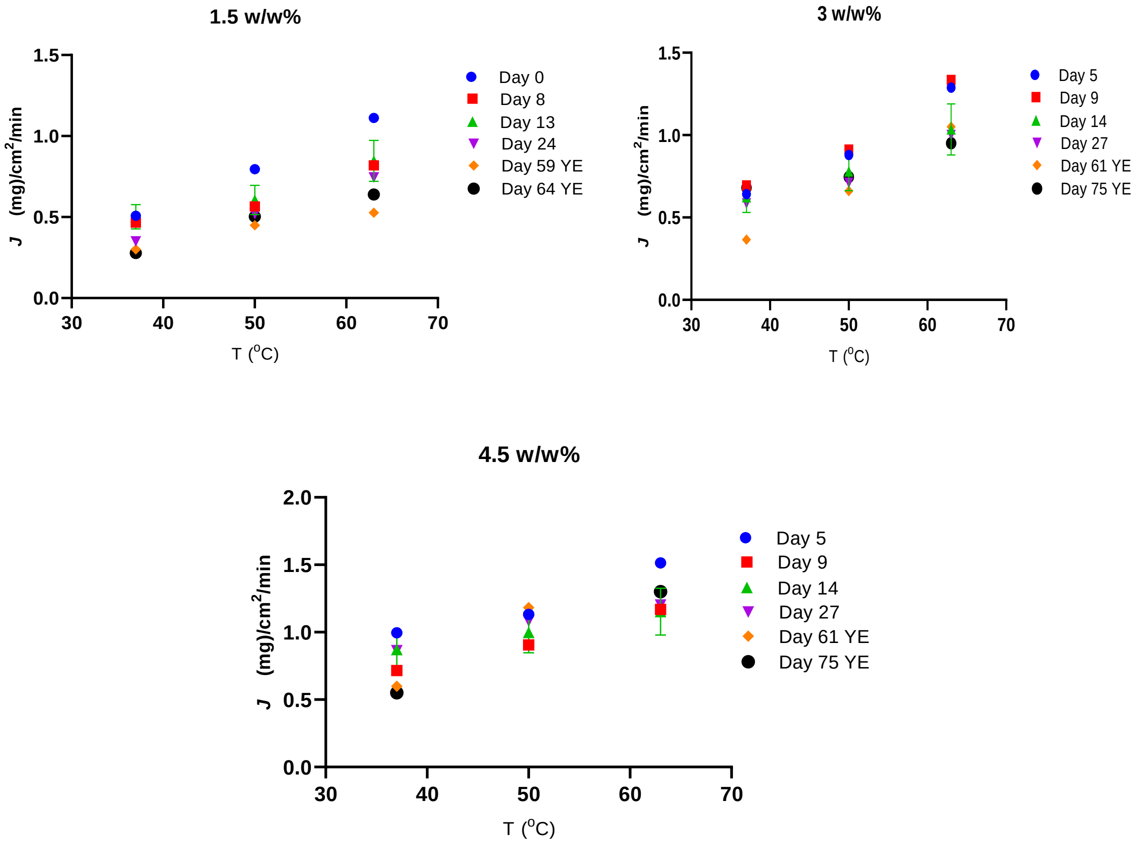

Figure S1. Total flux (*J*) at different temperatures as a function of operating temperature. YE is yeast extract at 200 mg / 100 mL water in the feed.

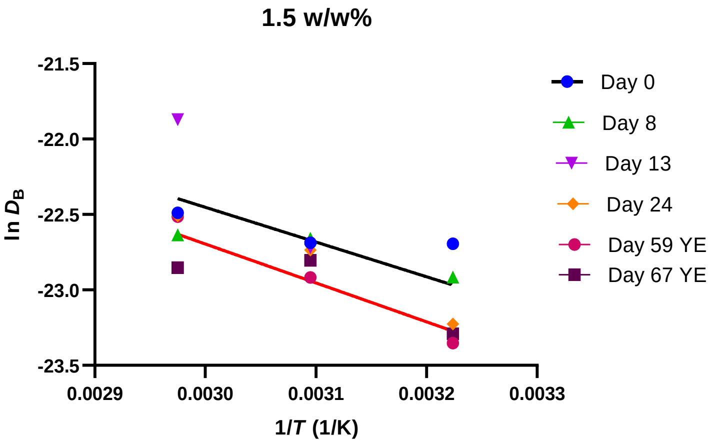

Figure S2. Temperature dependence of 1-butanol diffusivity in PTMSP. YE represents yeast extract dosed at 200 mg/100 mL of water in the feed. The black line represents Arrhenius-type model data for all feeds without yeast extract and red lines the model for feeds with yeast extract.

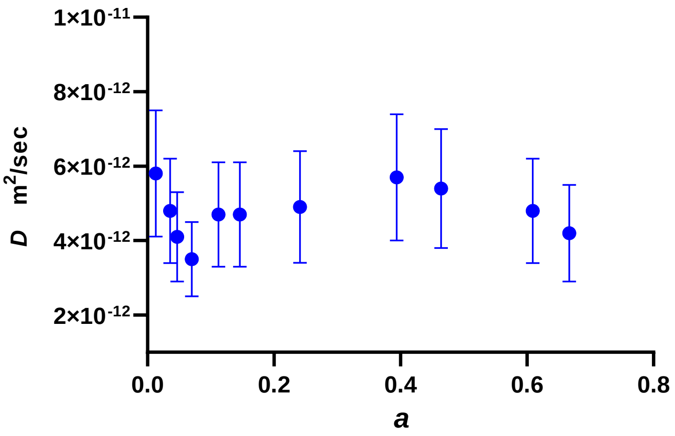

Figure S3. Diffusivity of 1-butanol vapors in PTMSP as measured with single vapor microgravimetry at 37 °C.

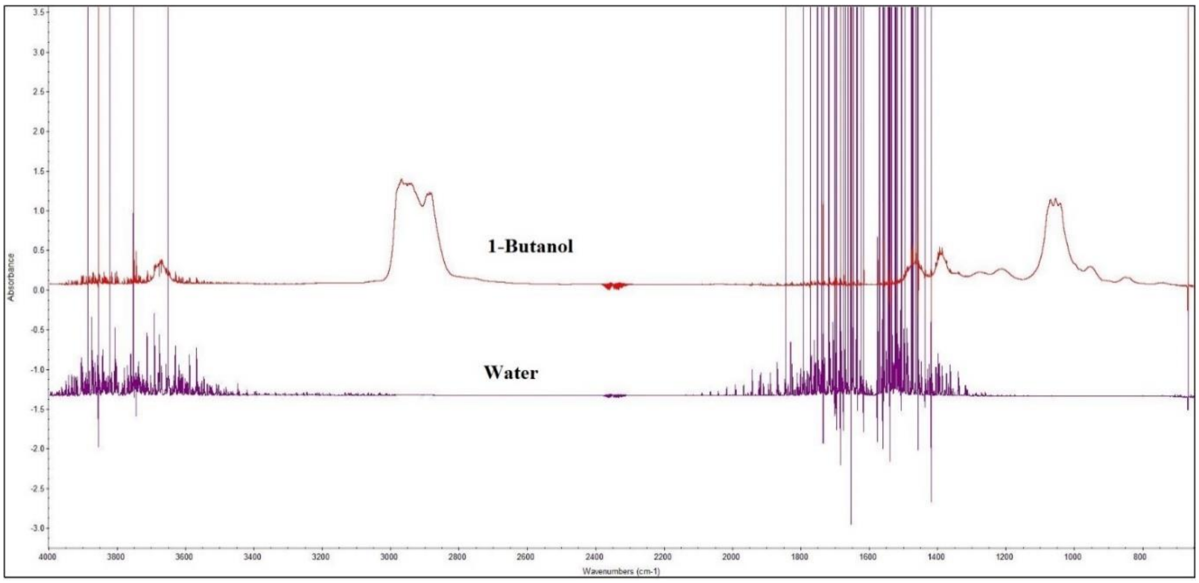

Figure S4. FTIR spectra for pure 1-butanol and water vapors. Bands from 1146.76 to 974.88 cm^{-1} were used to follow 1-butanol.

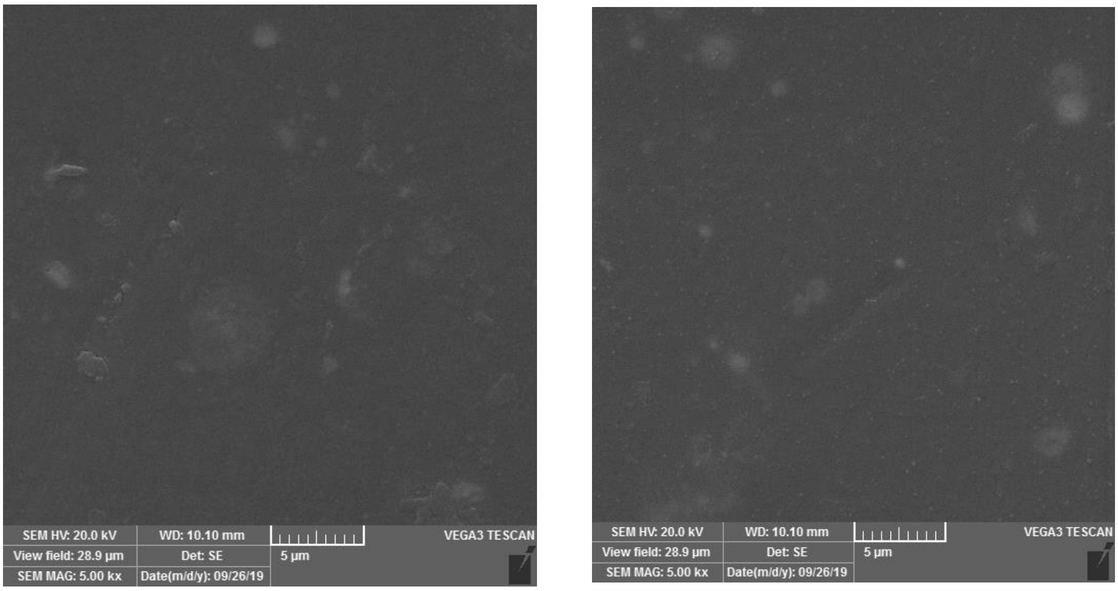

A B

Figure S5. Cross sectional scanning electron microscope images of aged membrane and fresh PTMSP membrane with high magnification 5000 times a) Aged b) fresh membrane.

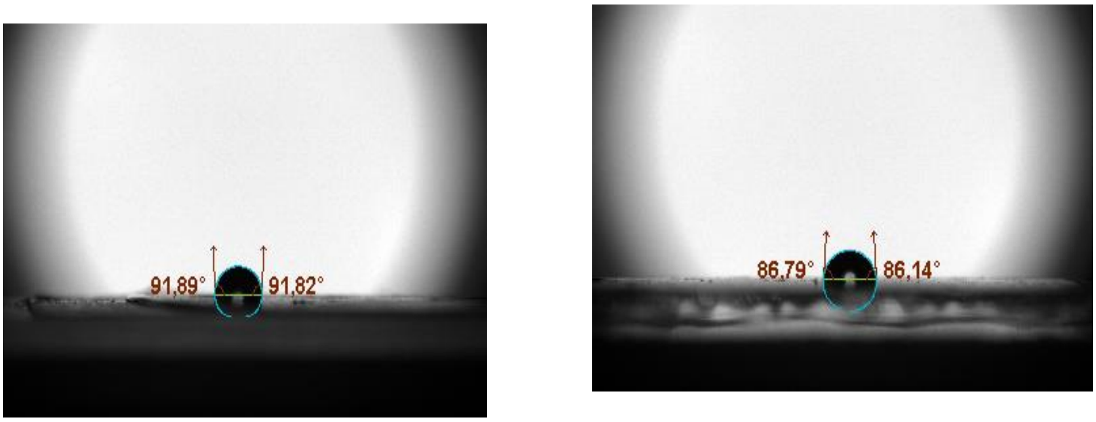

A B

Figure S6. Contact angle measurement images of fresh and aged membrane PTMSP membrane a) Fresh membrane b) aged membrane.
